# Editorial: Novel translational advances in artificial intelligence for diagnosis and treatment of cardiovascular diseases

**DOI:** 10.3389/fcvm.2025.1604528

**Published:** 2025-05-08

**Authors:** DeLisa Fairweather, Nay Aung, Rickey E. Carter

**Affiliations:** ^1^ Department of Cardiovascular Medicine, Mayo Clinic, Jacksonville, FL, United States; ^2^ Digital Environment Research Institute, Queen Mary University of London, London, United Kingdom; ^3^ Department of Quantitative Health Sciences, Mayo Clinic, Jacksonville, FL, United States

**Keywords:** artificial intelligence, large language models, cardiovascular disease, innovation, prediction models

**Editorial on the Research Topic**
Novel translational advances in artificial intelligence for diagnosis and treatment of cardiovascular diseases

Artificial Intelligence (AI) is poised to rapidly evolve medical practice through novel discoveries using deep learning (DL), large language models (LLM) and other forms of generative AI (1). These AI techniques are currently able to interpret and summarize data from immense data fields, where they can enhance image reconstruction, reduce noise, and assist in the interpretation of complex datasets (2).

There have been several pioneering AI applications in cardiology. Myocardial perfusion imaging (MPI), such as single photon emission computed tomography (SPECT) and positron emission tomography (PET), is used in disease diagnosis and risk assessment. AI application to SPECT or PET for coronary artery disease (CAD) has led to improved diagnostic accuracy, risk stratification, and therapeutic decision-making (2). Attia et al. developed an AI-enabled electrocardiograph (ECG) using a convolutional neural network that was able to detect the electrocardiographic signature of atrial fibrillation (AF) present during normal sinus rhythm using standard ECG leads (3). Another large study by Hannun et al., using a deep learning approach and ECG, found a similar successful detection ability for AF using AI which was better than the AF detection rate for physicians (4). A similar AI approach using an ECG was found to be highly successful at detecting dilated cardiomyopathy (DCM) (5). A randomized controlled trial conducted to examine whether AI-guided assessment of cardiomyopathy was similar or different to sonographers and cardiologists using echocardiography could not distinguish between the two methods, with the advantage that the AI-guided workflow saved time for sonographers and cardiologists (6). Interestingly, AI methods to detect cardiomyopathy have found that patients with so-called “false positives” are at greater risk of poor cardiovascular outcomes later, concluding that AI-models may be good at detecting potential cardiovascular issues in the future over current methods (7).

With these successes, AI is increasingly likely to be used to detect, diagnose and predict current and future cardiovascular events or poor outcomes. There are numerous impacts to incorporating AI into patient care including altering staffing levels, changing which equipment is used (i.e., ECG vs. echocardiogram), saving time, reducing costs, and improving outcomes. Additionally, AI is leading to major innovations in clinical practice. The possibilities are endless, with the potential to integrate multiple imaging technologies and interpret findings based on sex, race and biomarker data to improve prediction models (1).

The manuscripts included in this Research Topic provide further contributions to the field on this important topic (Table 1). A *Perspectives* article by Cerrato and Halamka, President and Senior Research Analyst of Mayo Clinic Platform, respectively, outline several examples of how AI is revolutionizing cardiovascular medicine and address AI's limitations. An article by Li and Ying describes the development of a comprehensive coronary heart disease (CHD) screening neural network model for patients with type 2 diabetes (T2D). The initial patient pool included 471 patients with CHD, among which 221 were also diagnosed with T2D (T2D-CHD), and 250 with CHD exclusively (CHD-only). Additionally, 228 patients with T2D but no CHD (T2D-only) were included for a comparative analysis. They found that the neural network model achieved an accuracy of 90.7%, recall of 90.78%, precision of 90.83%, and an F-1 score of 0.908. The logistic regression model demonstrated an accuracy of 90.13%, recall of 90.1%, precision of 90.22%, and an F-1 score of 0.9016. External validation reinforced the models' reliability and effectiveness in broader clinical settings. Similarly, Wang et al. describe the development of an Automated Machine Learning (AutoML) screening tool as a coronary artery disease (CAD) prediction model. They examined data from five distinct data sets for a total of 508 patients with CAD and 410 controls: Cleveland (303 observations), Hungary (294 observations), Switzerland (123 observations), VA Long Beach (200 observations), and Statlog (270 observations). They found that the AutoML model achieved an accuracy of 0.9167 and an AUC of 0.9562 in 4-fold cross-bagging and performed better than the individual baseline models in predicting CAD. A study by Li et al. used a contrastive learning model to refine its predictive capabilities for adverse outcomes from drug-eluting stent (DES) implantation. They used a large data set of 19,713 adult DES patients from the OneFlorida + Clinical Research Consortium for their analysis. Their approach demonstrated superior predictive performance for both ischemic and bleeding poor outcome events across prediction windows of 1, 2, 3, 6, and 12 months, with time-dependent concordance (Ctd) index values ranging from 0.88 to 0.80 and 0.82 to 0.77, respectively. The model consistently outperformed the baseline models, including DeepSurv, DeepHit, and SurvTrace, with statistically significant improvement in the Ctd-index values for most evaluated scenarios. Thus, all three of these studies found that the AI/deep learning models had relatively good accuracy at predicting the outcome that they were modeling. A review article in this Research Topic by Shyam-Sundar et al. described the primary imaging modalities used to detect acute myocarditis and performed a review of the literature for any studies examining AI/deep learning algorithms for acute myocarditis. They identified 6 studies in the literature that examined AI/deep learning methods to detect acute myocarditis. They summarized good predictive outcomes from several of the studies but also describe the limitations of AI for this condition. And finally, a *Perspectives* article by Adedinsewo et al. reflects on the authors' experiences conducting a randomized controlled trial (SPEC-AI Nigeria Trial) in Nigeria to examine the ability of AI to detect peripartum cardiomyopathy in pregnant women. Their trial showed AI-guided screening doubled the detection of cardiomyopathy (defined as left ventricular ejection fraction <50%) compared to usual care. Thus, the AI-screening tool appeared to be successful, but the authors describe the challenges and limitations involved in initiating this program and potential solutions. Overall, the articles in this Research Topic highlight the many approaches of AI to solving problems and improving cardiovascular care and health worldwide.

**Table 1:** Contributions to the research topic *“Novel translational advances in artificial intelligence for diagnosis and treatment of cardiovascular diseases”*.

Title	Authors	Article type	Topic	Patient data
How AI drives innovation in cardiovascular medicine.	Cerrato PL, Halamka JD	Perspective	Discusses the *potential and limitations* of AI and large language models.	–
A sensitivity indicator screening and intelligent classification method for the diagnosis of T2D-CHD.	Li J, Ying C	Original Research Article	Develop a comprehensive coronary heart disease (CHD) screening *neural network model* for type 2 diabetes (T2D) patients.	699 patients with CHD vs. 228 controls
Explainable coronary artery disease prediction model based on AutoGluon from AutoML framework.	Wang J, Xue Q, Zhang CWJ, Wong KKL, Liu Z	Original Research Article	Develop an *Automated Machine Learning (AutoML) screening tool* as a coronary artery disease (CAD) prediction model.	508 patients with CAD vs. 410 controls
Imaging for the diagnosis of acute myocarditis: Can artificial intelligence improve diagnostic performance?	Shyam-Sundar V, Harding D, Khan A, Abdulkareem M, Slabaugh G, Mohiddin SA, Petersen SE, Aung N	Review	Describes the role of cardiac magnetic resonance (CMR) imaging in the diagnosis of acute myocarditis, and a literature *review on the applications of AI and machine learning (ML)* to diagnose acute myocarditis.	6 studies identified
Contrastive learning with transformer for adverse endpoint prediction in patients on DAPT post-coronary stent implantation.	Li F, Sun Z, Abdelhameed A, Duan T, Rasmy L, Hu X, He J, Dang Y, Feng J, Li J, Wang Y, Lyu T, Braun N, Pham S, Gharacholou M, Fairweather D, Zhi D, Bian J, Tao C	Original Research Article	Applied *contrastive learning* to enable the model to refine its predictive capabilities for adverse outcomes from drug-eluting stent (DES) implantation by maximizing intra-class similarities and distinguishing inter-class differences. The model was holistically *optimized using multiple loss functions*, to ensure the predicted results closely aligned with the ground-truth values from various perspectives.	19,713 adult DES patients from the OneFlorida + Clinical Research Consortium
The role of artificial intelligence in aortic valve stenosis: a bibliometric analysis.	Chen S, Wu C, Zhang Z, Liu L, Zhu Y, Hu D, Jin C, Fu H, Wu J, Liu S	Bibliometric Analysis	The results highlight the growing impact of AI in aortic valve stenosis (AVS), particularly in cardiac imaging and predictive modeling. Core authors and institutions are primarily from the US and Germany.	118 articles analyzed
Contextual challenges in implementing artificial intelligence for healthcare in low-resource environments: insights from the SPEC-AI Nigeria trial.	Adedinsewo DA, Onietan D, Morales-Lara AC, Sheriff SM, Afolabi BB, Kushimo OA, Mbakwem AC, Ibiyemi KF, Ogunmodede JA, Raji HO, Ringim SH, Habib AA, Hamza SM, Ogah OS, Obajimi G, Saanu OO, Aborisade S, Jagun OE, Inofomoh FO, Adeolu T, Karaye KM, Gaya SA, Sa’ad Y, Alfa I, Yohanna C, Noseworthy PA, Carter RE, for the SPEC-AI Nigeria Investigators	Perspective	Perspectives on challenges in developing a randomized controlled clinical trial (NCT05438576) of an artificial intelligence (AI) technology in Nigeria to detect cardiomyopathy in obstetric patients.	–
